# KIF-2C expression is correlated with poor prognosis of operable esophageal squamous cell carcinoma male patients

**DOI:** 10.18632/oncotarget.11492

**Published:** 2016-08-22

**Authors:** Hao Duan, Xu Zhang, Fei-Xiang Wang, Mu-Yan Cai, Guo-Wei Ma, Hong Yang, Jian-Hua Fu, Zi-Hui Tan, Xia-Yu Fu, Qi-Long Ma, Xin-Ye Wang, Peng Lin

**Affiliations:** ^1^ Department of Thoracic Oncology, Sun Yat-Sen University Cancer Center, Guangzhou 510060, Guangdong Province, China; ^2^ Guangdong Esophageal Cancer Research Institute, Guangzhou 510060, Guangdong Province, China; ^3^ Department of Pathology, Sun Yat-Sen University Cancer Center, Guangzhou 510060, Guangdong Province, China; ^4^ Department of Thoracic Oncology, Cancer Center of Guangzhou Medical University, Guangzhou 510095, Guangdong Province, China

**Keywords:** esophageal squamous cell carcinoma, KIF-2C, prognosis

## Abstract

To determine the prognostic significance of Kinesin family member 2C (KIF-2C) expression in patients with operable esophageal squamous cell carcinoma (ESCC), we conducted an immunohistochemical analysis of KIF-2C expression in 415 surgically resected primary tumor tissues and 40 adjacent non-cancerous tissues from patients with operable ESCC. The median duration of postoperative follow-up was 76.0 months. Higher KIF-2C expression was associated with significantly increased risks of higher pathologic tumor (pT) status (P=0.038) and poorer tumor differentiation (P=0.022). For the entire cohort, KIF-2C expression was not an independent factor significantly associated with overall survival (OS) (P=0.097) or disease-free survival (DFS) (P=0.152). In female patients, KIF-2C expression had no effect on OS (P=0.880) and DFS (P=0.864). However, OS (hazard ratio (HR)=1.480, P=0.013) and DFS (HR=1.418, P=0.024) were worse for male patients with high KIF-2C expression compared with male patients with low KIF-2C expression. Moreover, the OS and DFS of male patients with high KIF-2C expression were also significantly shorter compared with female patients with low KIF-2C expression (P=0.022, P=0.029) and female patients with high KIF-2C expression (P=0.014, P=0.018). Based on these findings, KIF-2C expression in tumor tissues promises to serve as an independent prognostic marker for male, but not female, patients with operable ESCC. Prognosis was worse for male patients with high KIF-2C expression compared with patients with the same pathologic tumor-node-metastasis (pTNM) stage.

## INTRODUCTION

Patients with esophageal cancer have a dismal prognosis, and this disease caused approximately 455,800 deaths in 2012 worldwide [1, 2] with an incidence that was generally 3-4 times higher among men than women [1, 2]. Esophageal squamous cell carcinoma (ESCC) is a major pathologic type, which is prevalent in the highest-risk area stretching from northern Iran to north-central China [1, 2]. Tumor-node-metastasis (TNM) stage is the most commonly used prognostic model despite discrepant results within the same stage [3]. Therefore, to better inform the design of individualized treatment, there is an urgent demand for new parameters to determine risk stratification that complement TNM stage.

Most solid tumors are aneuploid and many contain aberrant chromosomes. For example, these chromosomal abnormalities are commonly caused by persistent abnormal chromosome bi-orientation and segregation during cell division mediated by microtubule dynamics [4–6]. The depolarization of microtubules by members of the kinesin-13 family is required to control microtubule dynamics [7, 8], and kinesin family member 2C (KIF-2C), also known as mitotic centromere-associated kinesin, is the most extensively characterized [9]. KIF-2C localizes to the cytoplasm throughout the cell cycle and is particularly enriched at centrosomes, centromeres/kinetochores, and the spindle midzone during mitosis [9–11] KIF-2C contributes to proper spindle formation [12–15], correction of aberrant attachments of microtubules to chromosomes, and chromosome segregation [10, 11, 16, 17]. The abnormal expression of KIF-2C is associated with abnormal mitosis, chromosomal aberrations, and malignant transformation [15, 17, 18]. Therefore, the deregulation of KIF-2C expression likely plays a role in cancer development and progression.

Although trace levels of KIF-2C are expressed in healthy organs, its levels are high in testis [18–20] as well as in colorectal cancer, gastric cancer, breast cancer, prostate cancer, and glioma [18–25]. Thus, its expression levels closely resemble those of cancer-testis antigens [26–28]. In patients with gastric cancer, colorectal cancer, and glioma, elevated KIF-2C expression serves as an independent marker of poor prognosis [22]. However, to the best of our knowledge, the prognostic significance of KIF-2C expression tumors of patients with ESCC patients is unknown. Therefore, the goal of the present study was to determine whether KIF-2C expression serves as a marker that will improve the management of patients with operable ESCC.

## RESULTS

### Immunohistochemical analysis of KIF-2C expression in tumor tissues and adjacent non-cancerous tissues

**Figure 1:**
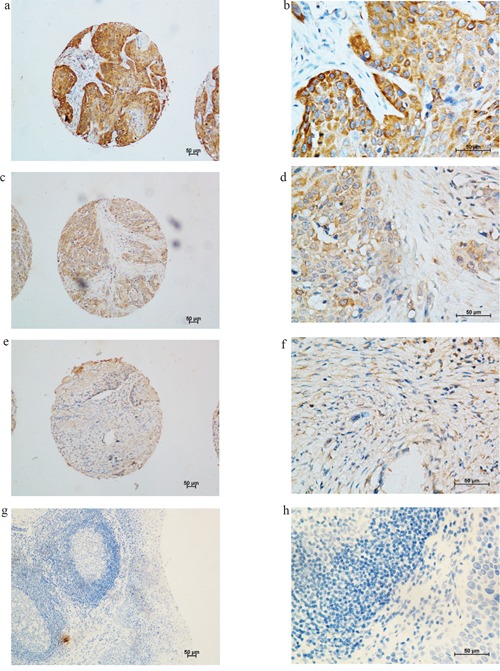
Immunohistochemical analysis of KIF-2C expression in tumor tissues and adjacent non-cancerous tissues of patients with esophageal squamous cell carcinoma (ESCC) Representative image of KIF-2C expression in the cytoplasm of cancer cells, but not in the non-cancerous cells. **a.** and **b.** strong staining of KIF-2C in cytoplasm of cancer cells a. 100×, b. 400×; **c.** and **d.** moderate staining of KIF-2C in cytoplasm of cancer cells c. 100×, d. 400×; **e.** and **f.** weak staining of KIF-2C in cytoplasm of cancer cells e. 100×, f. 400×; **g.** and **h.** negative KIF-2C staining in non-cancerous cells (negative control) g. 100×, h. 400×.

KIF-2C expression was observed in the cytoplasm of cancer cells (Figure 1a-1f), but not in the non-cancerous cells (Figure 1g-1h). KIF-2C was expressed in 99.5% of tumor cells, including 31.3%, 43.5%, and 24.8% that were weakly, moderately, and strongly positive. There were 215 patients (51.8% of patients) with expression scores ≥2.0 (high expression).

### Patients’ clinicopathologic characteristics

KIF-2C expression and clinicopathological characteristics did not differ significantly between eligible patients and those lost to follow-up. The median duration of postoperative follow-up was 76.0 months (interquartile range, 49.0–88.0 months). The median age of the patients was 57.0 years (range, 30.0–81.0 years). Median and mean survival times were 54.0 and 63.9 months, respectively. The 1-, 3-, and 5-year survival rates were 83.7%, 56.3%, 48.2%, respectively. Table 1 shows the details of patients’ clinicopathologic characteristics.

**Table 1:** Patients’ clinicopathologic features compared with KIF-2C expression

Variable	Cases, *n* (%)	KIF-2C, *n* (%)		P*
		<2.0	≥2.0	
Age (years)				0.165
≤ 57.0^†^	197(47.5)	102(51.0)	95(44.2)	
> 57.0	218(52.5)	98 (49.0)	120 (55.8)	
Sex				0.148
Male	308(74.2)	142 (71.0)	166 (77.2)	
Female	107 (25.8)	58 (29.0)	49 (22.8)	
Location				0.443
Upper	26 (6.3)	13 (6.5)	13 (6.0)	
Middle	286(68.9)	132(66.0)	154 (71.6)	
Lower	103 (24.8)	55 (27.5)	48 (22.3)	
Differentiation				**0.022**
Well	63 (15.2)	40 (20.0)	23 (10.7)	
Moderate	277(66.7)	129 (64.5)	148 (68.8)	
Poor	75 (18.1)	31 (15.5)	44 (20.5)	
pT status				**0.038**
T1	20 (4.8)	13 (6.5)	7 (3.3)	
T2	101 (24.3)	39 (19.5)	62 (28.8)	
T3	294(70.8)	148 (74.0)	146 (67.9)	
pN status				0.713
N0	227(54.7)	112 (56.0)	115 (53.5)	
N1	107(25.8)	53 (26.5)	54 (25.1)	
N2	65(15.7)	27 (13.5)	38 (17.7)	
N3	16(3.9)	8 (4.0)	8 (3.7)	
pTNM Stage				0.962
I	24 (5.8)	12 (6.0)	12 (5.6)	
II	233(56.1)	111 (55.5)	122 (56.7)	
III	158(38.1)	77 (38.5)	81 (37.7)	
Surgery			0.824	
Unqualified	135	64 (32.0)	71 (32.5)	
Qualified	280	136 (68.0)	144 (67.5)	
Total	415(100)	200(48.2)	215(51.8)	

pT, Pathologic tumor; pN, Pathologic node; Qualified surgery, R0 resection with at least 12 resected lymph nodes; Unqualified surgery, R0 resection with less than 12 resected lymph nodes.

* Chi-square test

† Median age

### Analysis of the association of KIF-2C expression with patients’ clinicopathological characteristics

Table 1 shows the distribution of patients’ clinicopathologic characteristics according to the levels of KIF-2C expression. Only tumor differentiation (*P*=0.022) and pathologic tumor (pT) status (*P*=0.038) were significantly associated with KIF-2C expression, and higher levels of KIF-2C were associated with poorer tumor differentiation or higher pT status.

### Analysis of the association between KIF-2C expression and prognosis

Kaplan–Meier analysis of the entire cohort revealed that sex, tumor differentiation, pT status, pathologic node (pN) status, pathologic TNM (pTNM) stage, surgical quality, and KIF-2C expression were associated with OS and DFS (all *P*<0.05) (Table 2). Multivariatable analysis of these factors indicated that male, higher pT and pN status, and unqualified surgery were adverse independent prognostic factors for OS and DFS (all *P*<0.05). However, the level of KIF-2C expression was not an independent prognostic factor for OS (*P*=0.097) or DFS (*P*=0.152) (Table 3).

**Table 2:** Kaplan-Meier analysis of factors associated with OS and DFS in the entire cohort

Variable	Mean OS (month)	Median OS (month)	*P*	Mean DFS (month)	Median DFS (month)	*P*
Age (year)			0.598			0.765
≤ 57.0^†^	65.6	54.0		59.7	40.0	
> 57.0	59.0	55.0		52.9	42.0	
Sex			**0.022**			**0.039**
Female	73.2	NR		67.2	74.0	
Male	58.7	42.0		52.9	34.0	
Location			0.128			0.267
Upper	67.8	79.0		51.8	35.0	
Middle	57.2	42.0		53.3	35.0	
Lower	71.2	NR		64.9	55.0	
Differentiation			**0.017**			**0.006**
Well	63.4	64.0		60.3	66.0	
Moderate	66.3	68.0		59.2	57.0	
Poor	44.6	26.0		38.9	22.0	
pT status			**0.004**			**0.006**
T1	58.2	NR		54.0	NR	
T2	71.1	74.0		67.4	77.0	
T3	59.1	40.0		51.9	29.0	
pN status			**< 0.001**			**< 0.001**
N0	80.1	NR		72.8	90.0	
N1	47.5	40.0		44.0	29.0	
N2	27.5	16.0		23.4	12.0	
N3	21.2	11.0		12.6	9.0	
pTNM Stage			**< 0.001**			**< 0.001**
I	67.1	NR		65.6	NR	
II	77.2	NR		69.7	83.0	
III	35.0	21.0		31.1	17.0	
Surgery			**0.013**			**0.046**
Unqualified	47.4	32.0		44.8	26.0	
Qualified	67.7	64.0		61.1	52.0	
KIF-2C			**0.036**			**0.038**
<2.0	68.9	71.0		62.5	59.0	
≥2.0	50.6	41.0		46.3	30.0	

pT, Pathologic tumor; pN, Pathologic node; OS, Overall survival; DFS, Disease-free survival; NR: Not reached; Qualified surgery, R0 resection with at least 12 resected lymph nodes; Unqualified surgery, R0 resection with less than 12 resected lymph nodes.

† Median age.

**Table 3:** Results of multivariable Cox regression analysis in the entire cohort

Prognostic Factor	Hazard ratio	95% CI	*P*
**OS**			
Sex (Male *vs.* Female)	1.566	1.126-2.179	**0.008**
Differentiation (Poor *vs.* Moderate *vs.* Well)	1.055	0.838-1.328	0.647
pT status (T3 *vs.* T2 *vs.* T1)	1.345	1.023-1.769	**0.034**
pN status (N3 *vs.* N2 *vs.* N1 *vs.*N0)	2.014	1.733-2.341	**< 0.001**
Surgery (Qualified *vs*. Unqualified)	0.539	0.404-0.720	**< 0.001**
KIF-2C (<2.0 *vs.* ≥2.0)	1.259	0.959-1.654	0.097
**DFS**			
Sex (Male *vs.* Female)	1.444	1.055-1.978	**0.022**
Differentiation (Poor *vs.* Moderate *vs.* Well)	1.108	0.885-1.387	0.369
pT status (T3 *vs.* T2 *vs.* T1)	1.307	1.006-1.698	**0.045**
pN status (N3 *vs.* N2 *vs.* N1 *vs.*N0)	1.984	1.711-2.300	**< 0.001**
Surgery (Qualified *vs*. Unqualified)	0.608	0.460-0.803	**< 0.001**
KIF-2C (<2.0 *vs.* ≥2.0)	1.213	0.931-1.579	0.152

CI: Confidence interval; pT, Pathologic tumor; pN, Pathologic node; OS, Overall survival; DFS, Disease-free survival; Qualified surgery, R0 resection with at least 12 resected lymph nodes; Unqualified surgery, R0 resection with less than 12 resected lymph nodes.

### Association between KIF-2C expression and prognosis according to patients’ sex

**Figure 2:**
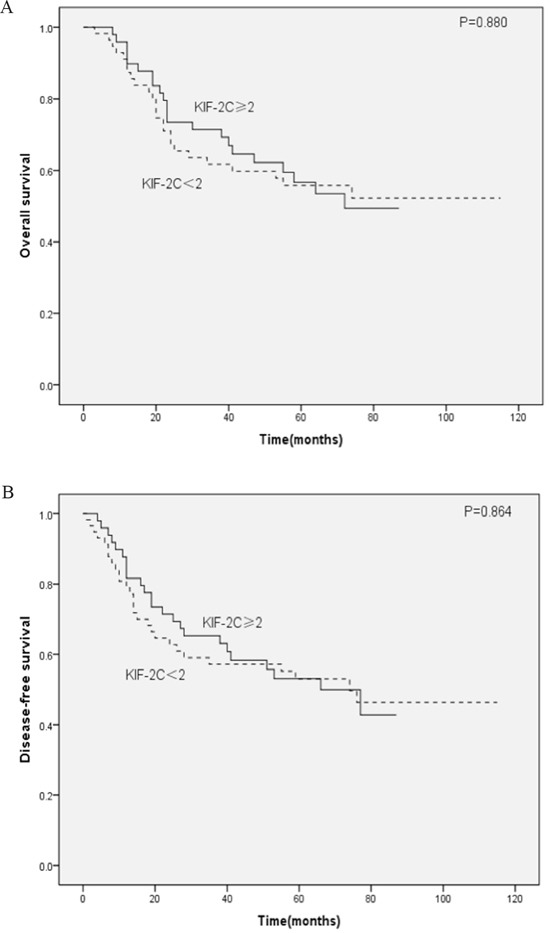
Kaplan–Meier analysis of female patients with ESCC **A.** Overall survival (OS) and **B.** disease-free survival (DFS) curves for female patients as a function of high or low KIF-2C expression.

**Figure 3:**
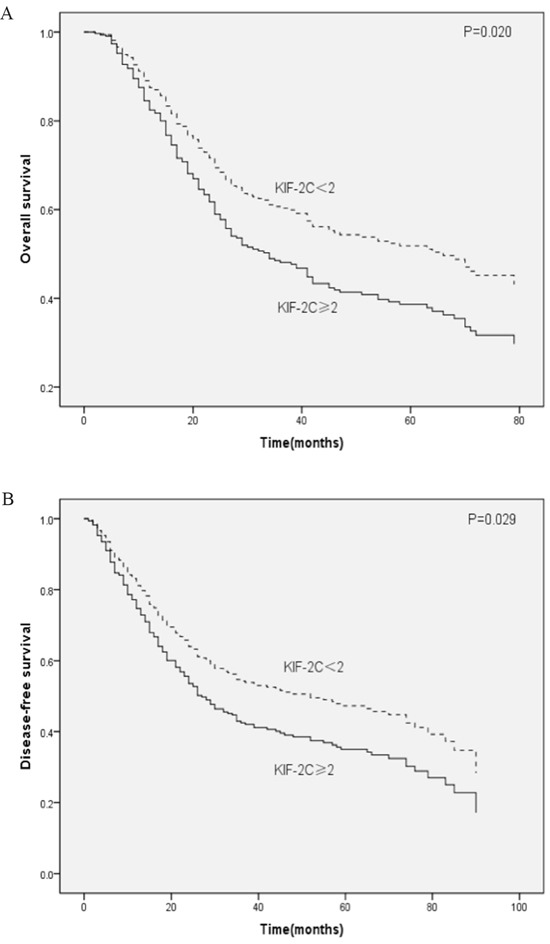
Kaplan–Meier analysis of male patients with ESCC **A.** OS and **B.** DFS curves for male patients with ESCC as a function of high or low KIF-2C expression.

Because KIF-2C is expressed highly in healthy testis [18–20], we speculate that KIF-2C need an appropriate environment consisting of androgen to carry out its function smoothly. The predictive significance of KIF-2C expression levels on OS and DFS were further assessed according to patients’ sex. The levels of KIF-2C expression of females were not significantly associated with OS (*P*=0.880) (Figure 2a) or DFS (P=0.864) (Figure 2b). Kaplan–Meier analysis of male patients (Table 4) revealed that the mean OS of the high-expression patients was 18.0 months shorter compared with that of the low-expression patients (P=0.017) (Figure 3a), and the mean DFS of the high-expression patients was 16.8 months shorter compared with that of the low-expression patients (P=0.016) (Figure 3b). Multivariable regression analysis of male patients indicated that high levels of KIF-2C were independently associated with a significantly increased risk of ESCC-related death (HR=1.480, *P*=0.013) and tumor recurrence (HR=1.418, *P*=0.024). Further, higher pT and pN status, and unqualified surgery were adverse independent prognostic factors for OS and DFS (all *P*<0.05) (Table 5).

**Table 4:** Kaplan–Meier analysis of factors associated with OS and DFS of male patients

Variable	Mean OS (month)	Median OS (month)	*P*	Mean DFS (month)	Median DFS (month)	*P*
Age (year)			0.867			0.867
≤ 57.0^†^	58.5	39.0		54.2	29.0	
> 57.0	57.3	45.0		51.0	39.0	
Location			0.183			0.595
Upper	69.0	79.0		56.4	58.0	
Middle	54.3	36.0		50.6	29.0	
Lower	62.8	54.0		56.3	42.0	
Differentiation			**0.009**			**0.006**
Well	62.5	70.0		61.3	66.0	
Moderate	61.4	56.0		54.4	39.0	
Poor	40.3	25.0		35.3	21.0	
pT status			**0.001**			**0.001**
T1	68.2	NR		63.2	NR	
T2	69.7	70.0		67.6	70.0	
T3	51.8	28.0		45.6	25.0	
pN status			**< 0.001**			**< 0.001**
N0	75.7	NR		69.6	85.0	
N1	45.3	36.0		42.0	26.0	
N2	25.3	16.0		21.0	12.0	
N3	15.0	11.0		10.1	6.0	
pTNM Stage			**< 0.001**			**< 0.001**
I	63.8	NR		63.3	NR	
II	71.8	NR		65.1	76.0	
III	32.6	20.0		29.3	15.0	
Surgery			**0.006**			**0.015**
Unqualified	42.9	25.0		40.1	22.0	
Qualified	63.1	58.0		57.6	44.0	
KIF-2C			**0.017**			**0.016**
<2.0	65.1	70.0		59.7	57.0	
≥2.0	47.1	29.0		42.9	25.0	

pT, Pathologic tumor; pN, Pathologic node; OS, Overall survival; DFS, Disease-free survival; NR: Not reached; Qualified surgery, R0 resection with at least 12 resected lymph nodes; Unqualified surgery, R0 resection with less than 12 resected lymph nodes.

† Median age.

**Table 5:** Results of multivariable Cox regression analysis of male patients

Prognostic Factor	Hazard ratio	95% CI	*P*
**OS**			
Differentiation (Poor *vs.* Moderate *vs.* Well)	1.075	0.821-1.408	0.600
pT status (T3 *vs.* T2 *vs.* T1)	1.591	1.130-2.240	**0.008**
pN status (N3 *vs.* N2 *vs.* N1 *vs.*N0)	2.174	1.818-2.599	**< 0.001**
Surgery (Qualified *vs*. Unqualified)	0.450	0.323-0.627	**< 0.001**
KIF-2C (<2.0 *vs.* ≥2.0)	1.480	1.085-2.020	**0.013**
**DFS**			
Differentiation (Poor *vs.* Moderate *vs.* Well)	1.141	0.876-1.487	0.329
pT status (T3 *vs.* T2 *vs.* T1)	1.575	1.131-2.193	**0.007**
pN status (N3 *vs.* N2 *vs.* N1 *vs.*N0)	2.142	1.800-2.548	**< 0.001**
Surgery (Qualified *vs*. Unqualified)	0.492	0.357-0.679	**< 0.001**
KIF-2C (<2.0 *vs.* ≥2.0)	1.418	1.048-1.918	**0.024**

CI: Confidence interval; pT, Pathologic tumor; pN, Pathologic node; OS, Overall survival; DFS, Disease-free survival; Qualified surgery, R0 resection with at least 12 resected lymph nodes; Unqualified surgery, R0 resection with less than 12 resected lymph nodes.

**Figure 4:**
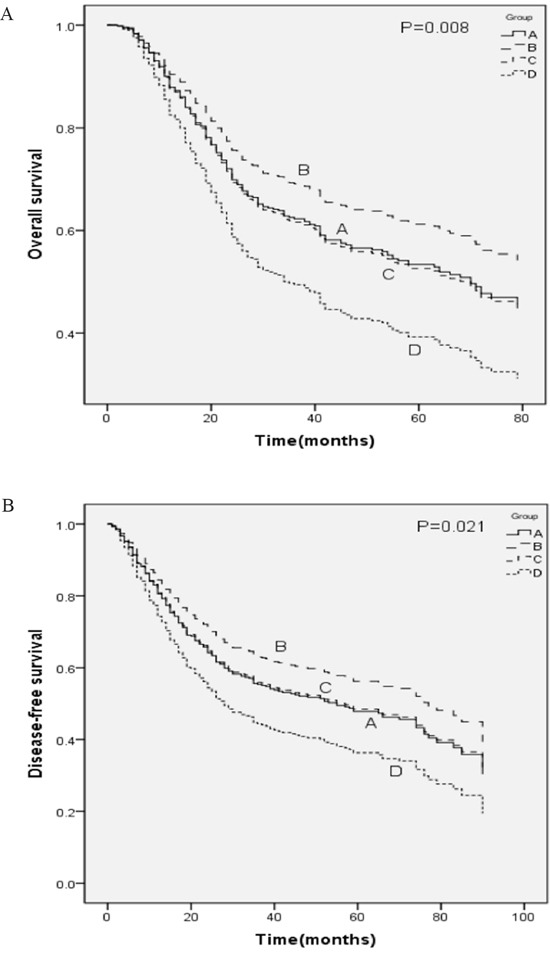
Kaplan–Meier analysis of groups A, B, C, and D **A.** OS and **B.** DFS curves of patients with ESCC according to the expression of KIF-2C and sex. Group A, female patients with KIF-2C <2.0; Group B, female patients with KIF-2C ≥2.0; Group C, male patients with KIF-2C <2.0; Group D, male patients with KIF-2C ≥2.0.

The prognostic significance of KIF-2C expression was further assessed after stratifying the complete cohort according to sex and KIF-2C expression. All patients were allocated into four groups as follows: A (female, low-expression), B (female, high-expression), C (male, low-expression), and D (male, high-expression). The numbers of patients in groups A–D described above were 58 (14%), 49 (11.8%), 142 (34.2%), and 166 (40%), respectively. Kaplan–Meier analysis revealed that the mean OS values (months) of patients in each group were 72.4, 60.0, 65.1, 47.1; and their mean DFS values (months) were 66.5, 55.5, 59.7, and 42.9, respectively. The OS and DFS of patients in group D were significantly shorter compared with those in group A (*P*=0.022, *P*=0.029), group B (*P*=0.014, *P*=0.018), and group C (*P*=0.017, *P*=0.016). In contrast, there were no significant differences in OS and DFS among patients in groups A, B, and C (all *P*>0.05) (Table 6, Figure 4a, 4b).

**Table 6:** Kaplan–Meier analysis of factors associated with OS and DFS of Groups A, B, C, and D

Groups	Mean OS (month)	Median OS (month)	*P*	Mean DFS (month)	Median DFS (month)	*P*
**Overall comparison**			**0.009**			**0.015**
A	72.4	NR		66.5	74.0	
B	60.0	72.0		55.5	66.0	
C	65.1	70.0		59.7	57.0	
D	47.1	29.0		42.9	25.0	
**Groups comparison**						
D *vs.* A	47.1-72.4	29.0-NR	**0.022**	42.9-66.5	25.0-74.0	**0.029**
D *vs.* B	47.1-60.0	29.0-72.0	**0.014**	42.9-55.5	25.0-66.0	**0.018**
D *vs.* C	47.1-65.1	29.0-70.0	**0.017**	42.9-59.7	25.0-57.0	**0.016**
C *vs.* A	65.1-72.4	70.0-NR	0.524	59.7-66.5	57.0-74.0	0.676
C *vs.* B	65.1-60.0	70.0-72.0	0.422	59.7-55.5	57.0-66.0	0.525
B *vs.* A	60.0-72.4	72.0-NR	0.880	55.5-66.5	66.0-74.0	0.864

Group A, Female patients with KIF-2C <2.0; Group B, Female patients with KIF-2C <2.0; Group C, Male patients with KIF-2C <2.0; Group D, Male patients with KIF-2C ≥2.0; OS, Overall survival; DFS, Disease-free survival; NR: Not reached.

## DISCUSSION

We conducted an immunohistochemical analysis of tumor tissues of 415 patients with operable ESCC. In this study, we did not detect a significant association between prognosis and the levels of expression of KIF-2C of the entire cohort. These findings differ from previous studies showing that elevated expression of KIF-2C serves as an independent prognostic marker for poor prognosis of unstratified patients with gastric and colorectal cancers as well as glioma [22–24]. Because KIF-2C is expressed highly in healthy testis, we reasoned that the association between prognosis and the levels of KIF-2C in tumor tissues might differ between male and female patients with ESCC. Interestingly, in the subgroup analyses conducted here, the prognoses of male patients with different levels of KIF-2C were significantly different, in contrast to those of female patients. Specifically, after controlling for tumor differentiation, pT and pN status, pTNM stage, and surgical quality, male patients with high levels of KIF-2C expression had significantly worse OS and DFS compared with male patients with low expression.

To further assess the effect of sex on the prognostic significance of KIF-2C expression, the complete cohort was divided into four groups according to sex and KIF-2C expression. Kaplan–Meier analysis suggested that the OS and DFS of male patients with high KIF-2C expression were significantly shorter compared with those of other patients.

To our knowledge, there is no published study that reports whether the role of KIF-2C protein on patients’ prognoses can be affected by the sex of patients [18–25]. However, from a relatively large cohort and extended follow-up, the present study suggests that male patients with high KIF-2C expression had a significantly worse prognosis. Indeed, the detailed mechanism that KIF-2C promotes tumor progression in male ESCC patients has not been revealed. However, the accumulating data suggest that reactivation of germline genes might confers essential characteristics to cancer cells [29]. Many germ cell proteins, including KIF-2C, are aberrantly expressed in cancer [30, 31]. The immature spermatogonia maintain proliferative capacity throughout their lives and continuously undergo meiosis which is comparable to the chromosomal changes observed in most cancers. It can be speculated that some meiotic programs are reactivated in cancer cells by the aberrantly expressed germ cell proteins and contribute to genome instability in cancer cells [32–34]. In this study, we found that male patients with high KIF-2C expression suffered the worst prognosis. As KIF-2C is highly expressed in testis, we speculate that an appropriate environment consisting of androgen is indispensable for KIF-2C to carry out its function smoothly. Therefore, the tumorigenic function of highly expressed KIF-2C in ESCC cells may also be facilitated with androgen in male patients.

Moreover, our findings indicated that higher levels of KIF-2C in tumor tissues were associated with an advanced malignant phenotype because of patients’ higher pT status and poorer tumor differentiation. Previous study showed that gastric cancer cells transfected with KIF-2C, but not controls, exhibited increased proliferation, migration, and decreased anoikis [24]. These phenomena correspond with the characteristics of poorly differentiated tumors and facilitate tumor invasion leading to higher pT status. Similarly, previous retrospective studies also detected an association between elevated levels of KIF-2C expression and lower histopathological grade of gliomas as well as higher tumor invasion in patients with gastric cancer and colorectal cancer [22–24]. Further, specific small interfering RNAs effectively suppressed KIF-2C expression and inhibited the growth of breast cancer and prostate cell lines that express high levels of KIF-2C [20, 21].

Such information will likely facilitate the development of individualized treatment. For example, the majority of the male patients in our study with high-expression of KIF-2C had shorter OS and DFS, indicating that surgical resection alone was insufficient. In healthy adults, KIF-2C expression is limited to male germ cells, but high expression can be observed in tumor cells [18–25]. However, the blood–testis barrier prevents immune cells from entering testis. Male germ cells also do not express HLA-class I molecules and cannot present antigens to T cells, allowing them to evade immunosurveillance by infiltrating T cells [35]. Therefore, KIF-2C may have antigenicity and the capacity to elicit strictly cancer-specific immune responses. Indeed, KIF-2C was capable of inducing spontaneous T cell responses in vivo resulting in frequently occurring and highly functional KIF2C-specific T cells in patients with colorectal cancer [18]. In addition, an inhibitor of KIF-2C induced the inhibition of responder T-lymphocyte activation [36, 37]. As a clear association between KIF-2C expression and cancer progression has been observed in this and previous studies [18–25], KIF-2C can be considered as an attractive target for immunotherapy.

There are some limitations of this study. For example, we conducted a retrospective study of patients treated at a single cancer center and just analyzed KIF-2C expression using immunohistochemistry. Future studies must be conducted to confirm these results and to identify the specific mechanism behind these phenomena.

In summary, this study showed that KIF-2C expression in tumor tissues promises to serve as an independent prognostic marker for male, but not female, patients with operable ESCC. Prognosis was worse for male patients with high KIF-2C expression compared with patients with the same pathologic tumor-node-metastasis (pTNM) stage.

## MATERIALS AND METHODS

### Patients

We conducted a retrospective review of the records of 481 consecutive patients with histologically confirmed, primary ESCC who were treated with curative-intent esophagectomy at the Cancer Center, Sun Yat-Sen University between October 2004 and May 2010. All patients underwent preoperative evaluation, including medical history, physical examination, esophageal barium swallow, endoscopic ultrasonography-aided tumor biopsy, and contrast-enhanced computed tomography (CT) of the neck, thorax, and upper abdomen. The exclusion criteria were as follows: (1) previous or another concomitant cancer; (2) neoadjuvant or adjuvant treatment; (3) non-R0 resection; (4) perioperative mortality; (5) stage 0, stage IV, or tumor pT4 disease; and (6) lack of insufficient or incomplete follow-up. Accordingly, data from 415 eligible patients were included in the present analysis.

The primary outcomes of the study were as follows: 1) overall survival (OS), defined as the time between surgery and cancer-caused death or the last investigation and 2) disease-free survival (DFS), which was defined as the time between the operation as well as local or distant soft tissue recurrence, or both, or the last investigation. The secondary outcomes were the associations of KIF-2C expression with clinicopathologic features. We collected clinicopathologic data including a patient's age, sex, tumor location, tumor differentiation, pT and pN status, pTNM stage, and surgical quality. One pathologist (M.Y.C.) conducted a second review of all pathological esophagectomy specimens. Tumor differentiation was determined according to the criteria proposed by the World Health Organization classification of Tumors of the Digestive System, Fourth Edition (2010). We staged patients according to the pTNM classification of the American Joint Committee on Cancer Staging Manual, 7th Edition [3]. The Medical Ethics Committee of the Cancer Center of Sun Yat-Sen University approved this study.

### Patient follow-up

Quarterly follow-up after esophagectomy performed at our institution is generally recommended for the first year, semiannually for the next two years, and annually thereafter for patients without evidence of recurrent disease. We routinely recorded the patient's medical history and conducted a physical examination as well as contrast-enhanced CT of the neck, thorax, and upper abdomen, including the liver and adrenals. The last follow-up examinations, which were conducted in December 2015, included verification of clinical attendance records and direct telecommunication with the patient or the family.

### Construction of tissue microarrays

We acquired a sample of a primary tumor from each of the 415 patients among whom 40 adjacent non-cancerous tissues were also collected. All samples were collected in the operating room and were routinely fixed immediately after collection in 10% neutral-buffered formalin for approximately 24 h at room temperature. After fixation, samples were dehydrated in xylene and embedded in paraffin (Oxford Labware, St. Louis, MO, USA). Two trained pathologists (M.Y.C., Y. L.) who were uninformed of the clinical data, diagnosed each primary tumor using hematoxylin-and-eosin stained slides, and the corresponding paraffin-embedded tissue blocks were acquired for further analysis.

Tissue microarrays were constructed using a Tissue Arrayer (Beecher Instruments, Sun Prairie, WI, USA). During sample selection, the pathologists marked areas on the paraffin wax tissue blocks containing tumors. For each case, three 1-mm tissue cores from the marked areas of the same tissue block were selected [38] and transferred to a tissue microarray. Hematoxylin-and eosin-stained sections from each tissue microarray block were checked by the pathologists to ensure that tumor and stromal tissues were included.

### Immunohistochemical methods

Immunohistochemical staining was performed using 4-μm sections that were obtained from tissue microarray blocks. Sections were reacted with a monoclonal antibody against KIF-2C (1:100 dilution, catalog number WH0011004M1; Sigma-Aldrich) overnight at 4°C. Immunoperoxidase staining was performed using an EliVison Plus (mouse/rabbit) IHC Kit (Maxim) according to the manufacturer's recommendations. Briefly, slides were incubated with polymer enhancer for 15 min and then with the same secondary antibody for 30 min at room temperature. After each step, slides were washed three times in phosphate-buffered saline solution. KIF-2C expression was defined as brown staining in the cytoplasm as recommend by the supplier. Nonimmune rabbit immunoglobulin G served as a negative control.

### Analysis of KIF-2C expression

Two pathologists (M.Y.C., Y. L.) independently scored all cases and then together, they reviewed the data. Using a modification of a published method [39–41], cytoplasmic expression of KIF-2C was scored using a semi-quantitative system. The intensity (I) and proportion (Prop) of staining in each core were recorded individually. Scores were calculated as follows: score = I × Prop, where I is the intensity of staining defined as no = 0, weak = 1, moderate = 2, and strong = 3, and Prop is the fraction of positive cells expressed as a percentage; and ≥200 nuclei were counted [39]. Therefore, this method yielded values from 0–3, and the final expression score of each case is expressed as the average score of three cores per tumor.

### Statistical analysis

All analyses were performed using the SPSS 20.0 software package (IBM Corp., Armonk, NY, USA). The cutoff value for KIF-2C expression was defined as 2.0 (median final expression score). High- and low-expression patients were defined as those of samples with final scores ≥2.0 or <2.0, respectively. All patients included in this study were treated with R0 resection without residual tumors at resection margin. Therefore, the surgical quality is mainly determined by the quality of lymphadenectomy. According to the recommendation of the American Joint Committee on Cancer Staging Manual, 7th Edition [3], a qualified lymphadenectomy for ESCC patients should involve at least 12 lymph nodes [42]. Therefore, we regarded 12 as the cutoff number of resected lymph nodes. In order to estimate the role of surgical quality, we divided the entire cohort into two groups. One named unqualified surgery group comprised patients whose resected lymph nodes were less than 12 (<12) and the other group named qualified surgery group comprised those whose resected lymph nodes were at least 12 (≥12). The correlations between KIF-2C expression and clinicopathologic characteristics were analyzed using the *χ*^2^ test. OS and DFS were estimated using the Kaplan–Meier method, and the statistical significance of the differences was assessed using the log-rank test. All significant parameters identified by Kaplan–Meier analysis were evaluated using with the Cox proportional hazards model. The significance of the association between predictors and survival was assessed according to hazard ratios (HRs) with 95% confidence intervals (CIs). *P*<0.05 (two-sided) were considered statistically significant. The statistical methods of this study were reviewed by Qing Liu at the Sun Yat-Sen University Cancer Center.

## References

[1] Torre LA, Bray F, Siegel RL, Ferlay J, Lortet-Tieulent J, Jemal A (2015). Global cancer statistics, 2012. CA Cancer J Clin.

[2] Enzinger PC, Mayer RJ (2003). Esophageal cancer. N Engl J Med.

[3] Rice TW, Blackstone EH, Rusch VW (2010). 7th edition of the AJCC Cancer Staging Manual: esophagus and esophagogastric junction. Ann Surg Oncol.

[4] Schvartzman JM, Sotillo R, Benezra R (2010). Mitotic chromosomal instability and cancer: mouse modelling of the human disease. Nat Rev Cancer.

[5] Bakhoum SF, Genovese G, Compton DA (2009). Deviant kinetochore microtubule dynamics underlie chromosomal instability. Curr Biol.

[6] Gadde S, Heald R (2004). Mechanisms and molecules of the mitotic spindle. Curr Biol.

[7] Walczak CE, Gayek S, Ohi R (2013). Microtubule-depolymerizing kinesins. Annu Rev Cell Dev Biol.

[8] Manning AL, Ganem NJ, Bakhoum SF, Wagenbach M, Wordeman L, Compton DA (2007). The kinesin-13 proteins Kif2a, Kif2b, and Kif2c/MCAK have distinct roles during mitosis in human cells. Mol Biol Cell.

[9] Wordeman L, Mitchison TJ (1995). Identification and partial characterization of mitotic centromere-associated kinesin, a kinesin-related protein that associates with centromeres during mitosis. J Cell Biol.

[10] Walczak CE, Mitchison TJ, Desai A (1996). XKCM1: a Xenopus kinesin-related protein that regulates microtubule dynamics during mitotic spindle assembly. Cell.

[11] Maney T, Hunter AW, Wagenbach M, Wordeman L (1998). Mitotic centromere-associated kinesin is important for anaphase chromosome segregation. J Cell Biol.

[12] Stout JR, Rizk RS, Kline SL, Walczak CE (2006). Deciphering protein function during mitosis in PtK cells using RNAi. BMC Cell Biol.

[13] Holmfeldt P, Stenmark S, Gullberg M (2004). Differential functional interplay of TOGp/XMAP215 and the KinI kinesin MCAK during interphase and mitosis. EMBO J.

[14] Ganem NJ, Compton DA (2004). The KinI kinesin Kif2a is required for bipolar spindle assembly through a functional relationship with MCAK. J Cell Biol.

[15] Cassimeris L, Morabito J (2004). TOGp, the human homolog of XMAP215/Dis1, is required for centrosome integrity, spindle pole organization, and bipolar spindle assembly. Mol Biol Cell.

[16] Andrews PD, Ovechkina Y, Morrice N, Wagenbach M, Duncan K, Wordeman L, Swedlow JR (2004). Aurora B regulates MCAK at the mitotic centromere. Dev Cell.

[17] Kline-Smith SL, Khodjakov A, Hergert P, Walczak CE (2004). Depletion of centromeric MCAK leads to chromosome congression and segregation defects due to improper kinetochore attachments. Mol Biol Cell.

[18] Gnjatic S, Cao Y, Reichelt U, Yekebas EF, Nolker C, Marx AH, Erbersdobler A, Nishikawa H, Hildebrandt Y, Bartels K, Horn C, Stahl T, Gout I (2010). NY-CO-58/KIF2C is overexpressed in a variety of solid tumors and induces frequent T cell responses in patients with colorectal cancer. Int J Cancer.

[19] Scanlan MJ, Welt S, Gordon CM, Chen YT, Gure AO, Stockert E, Jungbluth AA, Ritter G, Jager D, Jager E, Knuth A, Old LJ (2002). Cancer-related serological recognition of human colon cancer: identification of potential diagnostic and immunotherapeutic targets. Cancer Res.

[20] Shimo A, Tanikawa C, Nishidate T, Lin ML, Matsuda K, Park JH, Ueki T, Ohta T, Hirata K, Fukuda M, Nakamura Y, Katagiri T (2008). Involvement of kinesin family member 2C/mitotic centromere-associated kinesin overexpression in mammary carcinogenesis. Cancer Sci.

[21] Sircar K, Huang H, Hu L, Liu Y, Dhillon J, Cogdell D, Aprikian A, Efstathiou E, Navone N, Troncoso P, Zhang W (2012). Mitosis phase enrichment with identification of mitotic centromere-associated kinesin as a therapeutic target in castration-resistant prostate cancer. PLoS One.

[22] Bie L, Zhao G, Wang YP, Zhang B (2012). Kinesin family member 2C (KIF2C/MCAK) is a novel marker for prognosis in human gliomas. Clin Neurol Neurosurg.

[23] Ishikawa K, Kamohara Y, Tanaka F, Haraguchi N, Mimori K, Inoue H, Mori M (2008). Mitotic centromere-associated kinesin is a novel marker for prognosis and lymph node metastasis in colorectal cancer. Br J Cancer.

[24] Nakamura Y, Tanaka F, Haraguchi N, Mimori K, Matsumoto T, Inoue H, Yanaga K, Mori M (2007). Clinicopathological and biological significance of mitotic centromere-associated kinesin overexpression in human gastric cancer. Br J Cancer.

[25] Shimo A, Nishidate T, Ohta T, Fukuda M, Nakamura Y, Katagiri T (2007). Elevated expression of protein regulator of cytokinesis 1, involved in the growth of breast cancer cells. Cancer Sci.

[26] Gure AO, Chua R, Williamson B, Gonen M, Ferrera CA, Gnjatic S, Ritter G, Simpson AJ, Chen YT, Old LJ, Altorki NK (2005). Cancer-testis genes are coordinately expressed and are markers of poor outcome in non-small cell lung cancer. Clin Cancer Res.

[27] Chen YT, Scanlan MJ, Sahin U, Tureci O, Gure AO, Tsang S, Williamson B, Stockert E, Pfreundschuh M, Old LJ (1997). A testicular antigen aberrantly expressed in human cancers detected by autologous antibody screening. Proc Natl Acad Sci U S A.

[28] Tureci O, Sahin U, Zwick C, Koslowski M, Seitz G, Pfreundschuh M (1998). Identification of a meiosis-specific protein as a member of the class of cancer/testis antigens. Proc Natl Acad Sci U S A.

[29] Hanahan D, Weinberg RA (2011). Hallmarks of cancer: the next generation. Cell.

[30] Silva WA, Gnjatic S, Ritter E, Chua R, Cohen T, Hsu M, Jungbluth AA, Altorki NK, Chen YT, Old LJ, Simpson AJ, Caballero OL (2007). PLAC1, a trophoblast-specific cell surface protein, is expressed in a range of human tumors and elicits spontaneous antibody responses. Cancer Immun.

[31] Acevedo HF, Tong JY, Hartsock RJ (1995). Human chorionic gonadotropin-beta subunit gene expression in cultured human fetal and cancer cells of different types and origins. Cancer.

[32] Erenpreisa J, Cragg MS (2010). MOS, aneuploidy and the ploidy cycle of cancer cells. Oncogene.

[33] Ianzini F, Kosmacek EA, Nelson ES, Napoli E, Erenpreisa J, Kalejs M, Mackey MA (2009). Activation of meiosis-specific genes is associated with depolyploidization of human tumor cells following radiation-induced mitotic catastrophe. Cancer Res.

[34] Kalejs M, Ivanov A, Plakhins G, Cragg MS, Emzinsh D, Illidge TM, Erenpreisa J (2006). Upregulation of meiosis-specific genes in lymphoma cell lines following genotoxic insult and induction of mitotic catastrophe. BMC Cancer.

[35] Janitz M, Fiszer D, Michalczak-Janitz K, Lukaszyk A, Fernandez N, Skorupski W, Kurpisz M (1994). Analysis of mRNA for class I HLA on human gametogenic cells. Mol Reprod Dev.

[36] Matsumoto Y, Sahara H, Fujita T, Shimozawa K, Takenouchi M, Torigoe T, Hanashima S, Yamazaki T, Takahashi S, Sugawara F, Mizushina Y, Ohta K, Takahashi N (2002). An immunosuppressive effect by synthetic sulfonolipids deduced from sulfonoquinovosyl diacylglycerols of sea urchin. Transplantation.

[37] Aoki S, Ohta K, Yamazaki T, Sugawara F, Sakaguchi K (2005). Mammalian mitotic centromere-associated kinesin (MCAK): a new molecular target of sulfoquinovosylacylglycerols novel antitumor and immunosuppressive agents. FEBS J.

[38] Kononen J, Bubendorf L, Kallioniemi A, Barlund M, Schraml P, Leighton S, Torhorst J, Mihatsch MJ, Sauter G, Kallioniemi OP (1998). Tissue microarrays for high-throughput molecular profiling of tumor specimens. Nat Med.

[39] Gomez-Roca C, Raynaud CM, Penault-Llorca F, Mercier O, Commo F, Morat L, Sabatier L, Dartevelle P, Taranchon E, Besse B, Validire P, Italiano A, Soria JC (2009). Differential expression of biomarkers in primary non-small cell lung cancer and metastatic sites. J Thorac Oncol.

[40] Schoppmann SF, Schindl M, Bayer G, Aumayr K, Dienes J, Horvat R, Rudas M, Gnant M, Jakesz R, Birner P (2003). Overexpression of Id-1 is associated with poor clinical outcome in node negative breast cancer. Int J Cancer.

[41] Luo KJ, Wen J, Xie X, Fu JH, Luo RZ, Wu QL, Hu Y (2012). Prognostic relevance of Id-1 expression in patients with resectable esophageal squamous cell carcinoma. Ann Thorac Surg.

[42] Dutkowski P, Hommel G, Bottger T, Schlick T, Junginger T (2002). How many lymph nodes are needed for an accurate pN classification in esophageal cancer? Evidence for a new threshold value. Hepatogastroenterology.

